# Supplementary material for: Genome-wide cross-cancer analysis illustrates the critical role of bimodal miRNA in patient survival and drug responses to PI3K inhibitors
Source: PLoS Comput Biol. 2022 May 31;18(5):e1010109. doi: 10.1371/journal.pcbi.1010109 (PMC9187341; doi:10.1371/journal.pcbi.1010109)
Supplement: S1 Table — Pairwise Pearson correlations were calculated between miRNA with bimodality index > 1.4. Reported modules contain at least three miRNA with correlations > 0.5 between all miRNA pairs. (PDF) [file pcbi.1010109.s001.pdf]

Table S1. Concurrently expressed bimodal miRNA modules.
Pairwise Pearson correlations were calculated between miRNA with bimodality index > 1.4. Reported modules contain at least three miRNA with correlations > 0.5 between all miRNA pairs.

Cancer	Module	miRNA
Breast	1	hsa-mir-616<br>hsa-mir-628<br>hsa-mir-3605
Head and Neck	1	hsa-mir-9-1<br>hsa-mir-9-2<br>hsa-mir-9-3
	2	hsa-mir-105-1<br>hsa-mir-105-2<br>hsa-mir-767
	3	hsa-mir-1-1<br>hsa-mir-1-2<br>hsa-mir-133a-1<br>hsa-mir-133a-2<br>hsa-mir-133b<br>hsa-mir-206
Kidney	1	hsa-mir-96<br>hsa-mir-182<br>hsa-mir-183
	2	hsa-mir-190a<br>hsa-mir-192<br>hsa-mir-194-1<br>hsa-mir-194-2<br>hsa-mir-215
	3	hsa-mir-126<br>hsa-mir-143<br>hsa-mir-145<br>hsa-mir-195<br>hsa-mir-224<br>hsa-mir-452<br>hsa-mir-497
	4	hsa-mir-122<br>hsa-mir-126<br>hsa-mir-151b<br>hsa-mir-210<br>hsa-mir-215<br>hsa-mir-628<br>hsa-mir-1271<br>hsa-mir-3605
	5	hsa-mir-16-1<br>hsa-mir-16-2<br>hsa-mir-26b<br>hsa-mir-126

	[illegible]	hsa-mir-628<br>hsa-mir-1271<br>hsa-mir-2355<br>hsa-mir-3612
Liver	1	hsa-mir-199a-1<br>hsa-mir-199a-2<br>hsa-mir-199b<br>hsa-mir-214<br>hsa-mir-708
	2	hsa-mir-105-1<br>hsa-mir-105-2<br>hsa-mir-767<br>hsa-mir-4652
	3	hsa-mir-96<br>hsa-mir-182<br>hsa-mir-183
	4	hsa-mir-216a<br>hsa-mir-216b<br>hsa-mir-217
Lung	1	hsa-mir-9-1<br>hsa-mir-9-2<br>hsa-mir-9-3
	2	hsa-mir-105-1<br>hsa-mir-105-2<br>hsa-mir-767
	3	hsa-mir-143<br>hsa-mir-205<br>hsa-mir-944<br>hsa-mir-6499
Prostate	N/A	N/A
Stomach	1	hsa-mir-1-1<br>hsa-mir-133a-1<br>hsa-mir-133a-2<br>hsa-mir-133b<br>hsa-mir-143<br>hsa-mir-490
	2	hsa-mir-105-1<br>hsa-mir-105-2<br>hsa-mir-767
	1	hsa-mir-19a<br>hsa-mir-20a<br>hsa-mir-93
		hsa-mir-136<br>hsa-mir-154<br>hsa-mir-337<br>hsa-mir-369<br>hsa-mir-376c

Thyroid	2	hsa-mir-381<br>hsa-mir-382<br>hsa-mir-409<br>hsa-mir-485<br>hsa-mir-493<br>hsa-mir-675<br>hsa-mir-758<br>hsa-mir-889<br>hsa-mir-1247
Uterus	1	hsa-mir-34b<br>hsa-mir-34c<br>hsa-mir-449a<br>hsa-mir-449b<br>hsa-mir-449c
	2	hsa-mir-9-1<br>hsa-mir-9-2<br>hsa-mir-9-3
