# Supplementary material for: Genome-wide cross-cancer analysis illustrates the critical role of bimodal miRNA in patient survival and drug responses to PI3K inhibitors
Source: PLoS Comput Biol. 2022 May 31;18(5):e1010109. doi: 10.1371/journal.pcbi.1010109 (PMC9187341; doi:10.1371/journal.pcbi.1010109)
Supplement: S2 Table — Hierarchical clustering was performed using bimodal miRNA co-expression modules, and patients were divided into two groups. Using the two groups, a Cox proportional hazards regression was fit for each cancer type. The reported hazard ratio (HR) denotes the risk of death in Group 2 compared to Group 1. The statistical significance (p-value) of each HR was determined using a Wald test. MST: median survival time, CI: confidence interval. (PDF) [file pcbi.1010109.s002.pdf]

Cancer	Group 1 MST in Days (95% CI)	Group 2 MST in Days (95% CI)	HR (95% CI)	p-value
Breast	6593 (3461-N/A)	3945 (3472-N/A)	0.86 (0.62-1.19)	0.36
Head and Neck	2002 (1718-2900)	915 (602-1430)	1.85 (1.42-2.42)	5.21E-06
Kidney	N/A	3554 (2564-N/A)	1.39 (1.05-1.85)	0.02
Liver	848 (757-1791)	2456 (1852-N/A)	0.46 (0.32-0.65)	1.10E-05
Lung	1423 (1067-2284)	1600 (1357-1798)	0.87 (0.69-1.09)	0.23
Stomach	779 (513-N/A)	1153 (792-N/A)	0.74 (0.54-1.03)	0.077
Thyroid	N/A	N/A	0.32 (0.12-0.86)	0.025
Uterus	N/A (3112-N/A)	N/A (3423-N/A)	0.62 (0.40-0.97)	0.034

Table S2. Survival analysis using multiple bimodal miRNA modules. Hierarchical clustering was performed using bimodal miRNA co-expression modules, and patients were divided into two groups. Using the two groups, a Cox proportional hazards regression was fit for each cancer type. The reported hazard ratio (HR) denotes the risk of death in Group 2 compared to Group 1. The statistical significance (p-value) of each HR was determined using a Wald test. MST: median survival time, CI: confidence interval.
