# Supplementary material for: Genome-wide cross-cancer analysis illustrates the critical role of bimodal miRNA in patient survival and drug responses to PI3K inhibitors
Source: PLoS Comput Biol. 2022 May 31;18(5):e1010109. doi: 10.1371/journal.pcbi.1010109 (PMC9187341; doi:10.1371/journal.pcbi.1010109)
Supplement: S3 Table — Values represent correlations between miRNA expression and drug log(IC50) value. (PDF) [file pcbi.1010109.s003.pdf]

Table S3. Drug sensitivity based on miR-105 and miR-767 expression. Values represent correlations between miRNA expression and drug log(IC50) value.

Drug	miR-105	miR-767
AKT inhibitor VIII	-0.82	-0.81
Idelalisib	-0.76	-0.77
PD-0332991	-0.76	-0.72
PIK-93	-0.67	0.44
Omipalisib	-0.63	0.09
ZSTK474	-0.62	-0.61
KIN001-244	-0.61	0.48
GSK690693	-0.61	0.58
AICA Ribonucleotide	-0.61	-0.64
Pictilisib	-0.60	-0.56
SB52334	-0.59	0.56
Pictilisib	-0.53	-0.55
Tamoxifen	-0.52	-0.59
AZD8055	-0.52	-0.52
OSI-027	-0.50	0.22
AZD6482	-0.50	-0.48
PLX4720	-0.50	-0.47
MK-2206	-0.49	-0.48
AKT inhibitor VIII	-0.48	-0.51
Palbociclib	-0.48	-0.50
AS605240	-0.48	-0.44
YM201636	-0.48	0.55
JW-7-24-1	-0.46	-0.46
QL-X-138	-0.43	-0.03
CUDC-101	-0.42	-0.42
Dacinostat	-0.37	-0.33
CP724714	-0.37	-0.42
NG-25	-0.34	-0.32
UNC0638	-0.33	-0.28
AR-42	-0.33	-0.32
Selumetinib	-0.33	-0.34
UNC0638	-0.32	-0.27
Avagacestat	-0.32	-0.34
MPS-1-IN-1	-0.30	0.53
JQ1	-0.30	-0.33
PD-0325901	-0.29	-0.29
Belinostat	-0.28	-0.27
Shikonin	-0.28	-0.37
SB505124	-0.28	-0.34
AZD6482	-0.26	-0.32
Quizartinib	-0.26	-0.30
GW441756	-0.26	-0.39

NSC-207895	-0.26	-0.26
XAV939	-0.25	-0.24
Afatinib	-0.25	-0.24
Pelitinib	-0.23	0.48
KIN001-266	-0.23	0.50
BIX02189	-0.22	0.59
Tubastatin A	-0.19	-0.21
AZD6244	-0.18	-0.17
5-Fluorouracil	-0.18	-0.16
TPCA-1	-0.17	0.69
Masitinib	-0.16	0.10
NU7441	-0.15	-0.15
CAY10603	-0.14	0.29
Temsirolimus	-0.14	-0.14
THZ-2-102-1	-0.14	0.11
CCT007093	-0.14	-0.21
Afatinib	-0.13	-0.12
QL-XII-47	-0.11	-0.11
PFI-3	-0.11	-0.16
THZ-2-49	-0.10	0.12
CP466722	-0.10	-0.09
Bicalutamide	-0.10	-0.10
Nilotinib	-0.10	-0.07
TL-2-105	-0.09	-0.18
Cabozantinib	-0.09	-0.11
PHA-793887	-0.08	0.45
TAE684	-0.08	-0.03
WZ3105	-0.06	-0.04
SNX-2112	-0.06	0.35
BMS-536924	-0.06	-0.10
Genentech Cpd 10	-0.05	-0.08
XMD13-2	-0.05	0.46
Elesclomol	-0.05	-0.08
Docetaxel	-0.05	-0.09
Serdemetan	-0.04	-0.03
Paclitaxel	-0.04	0.04
Rucaparib	-0.03	-0.10
Topotecan	-0.03	0.01
PI-103	-0.03	0.09
17-AAG	-0.03	0.01
I-BET-762	-0.01	0.34
Foretinib	0.00	0.32
Nutlin-3	0.00	0.00
BMS-754807	0.01	0.07
Fedratinib	0.02	0.34
NVP-BHG712	0.03	-0.11
GSK269962A	0.03	-0.01

**FR-180204**	0.04	0.11
**PF-4708671**	0.04	-0.02
**EHT-1864**	0.06	-0.01
**PLX-4720**	0.09	0.15
**VX-11e**	0.09	0.07
**Bryostatin 1**	0.09	0.01
**PLX-4720**	0.09	0.17
**NPK76-II-72-1**	0.10	0.12
**Linsitinib**	0.11	0.18
**Daporinad**	0.11	0.07
**XMD14-99**	0.11	0.08
**Refametinib**	0.12	0.13
**LBW242**	0.12	0.15
**Erlotinib**	0.12	0.15
**ZD-6474**	0.12	0.15
**Dactolisib**	0.13	0.11
**FTI-277**	0.13	0.20
**Vorinostat**	0.14	0.08
**KIN001-236**	0.17	0.20
**PHA-665752**	0.17	0.20
**Trametinib**	0.17	0.18
**KIN001-260**	0.18	0.32
**L-685458**	0.18	0.21
**TL-1-85**	0.18	0.21
**Ispinesib Mesylate**	0.18	0.20
**KU-55933**	0.19	0.16
**Sorafenib**	0.20	0.23
**ZM447439**	0.20	0.17
**AZD0530**	0.20	0.24
**AT-7519**	0.20	0.22
**Panobinostat**	0.20	0.22
**RAF265**	0.21	0.26
**PF2341066**	0.21	0.24
**PAC-1**	0.21	0.25
**Refametinib**	0.21	0.21
**Lapatinib**	0.23	0.28
**Enzastaurin**	0.24	0.26
**AEW541**	0.25	0.28
**Tanespimycin**	0.27	0.25
**BX-912**	0.28	0.22
**Nutlin-3a (-)**	0.28	0.23
**BAY-61-3606**	0.29	0.25
**Methotrexate**	0.29	0.32
**KIN001-270**	0.29	0.32
**CI-1040**	0.30	0.29
**FMK**	0.31	0.29
**Alectinib**	0.31	-0.22

GSK1070916	0.31	0.25
IPA-3	0.31	0.24
Dabrafenib	0.32	0.36
Vinblastine	0.32	0.33
TKI258	0.32	0.37
T0901317	0.33	-0.06
NSC-87877	0.33	0.32
SB590885	0.33	0.40
Avagacestat	0.34	0.33
JQ1	0.35	0.42
Etoposide	0.35	0.26
Doxorubicin	0.36	0.34
Irinotecan	0.36	0.38
RO-3306	0.36	0.32
CX-5461	0.37	0.19
Amuvatinib	0.37	-0.17
BMS-345541	0.37	0.37
Piperlongumine	0.37	0.28
(5Z)-7-Oxozeaenol	0.38	0.35
PD0325901	0.39	0.41
Sepantronium bromide	0.40	0.44
Selumetinib	0.41	0.41
Obatoclax Mesylate	0.41	0.36
TW 37	0.42	0.42
Zibotentan	0.45	0.37
Bosutinib	0.45	0.49
Mitomycin-C	0.46	0.46
Tipifarnib	0.46	0.45
JQ12	0.46	0.39
CCT-018159	0.48	0.42
TAK-715	0.49	0.43
XMD15-27	0.50	0.22
Pazopanib	0.50	0.55
SB216763	0.50	0.45
rTRAIL	0.50	0.47
VX-702	0.54	0.49
Phenformin	0.54	0.54
Tretinoin	0.55	0.53
Epothilone B	0.55	0.56
Bleomycin (50 uM)	0.55	0.49
PFI-1	0.56	0.63
YK-4-279	0.57	0.59
STF-62247	0.58	0.59
Pevonedistat	0.58	0.59
UNC1215	0.58	0.55
OSU-03012	0.59	0.59
Ruxolitinib	0.59	0.50

Vismodegib	0.59	0.57
Vinorelbine	0.59	0.62
Motesanib	0.59	0.58
Cetuximab	0.59	0.62
FH535	0.60	0.57
JNK-9L	0.60	0.59
Nilotinib	0.60	0.59
Y-39983	0.61	0.35
SL0101	0.61	0.58
Navitoclax	0.61	0.54
Luminespib	0.61	0.61
GSK650394	0.62	0.60
Linifanib	0.62	-0.19
IOX2	0.62	0.62
SN-38	0.63	0.56
ICL1100013	0.64	0.66
Olaparib	0.64	0.58
AS601245	0.65	0.65
AZD7762	0.65	0.70
Veliparib	0.66	0.64
QL-XI-92	0.66	0.10
GSK1904529A	0.67	0.74
Cisplatin	0.67	0.62
OSI-930	0.68	0.08
Gemcitabine	0.69	0.72
WHI-P97	0.69	0.27
Bexarotene	0.70	0.75
Axitinib	0.70	0.64
Ponatinib	0.70	0.69
CHIR-99021	0.71	0.72
Temozolomide	0.71	0.66
DMOG	0.71	0.77
Lenalidomide	0.71	0.69
GSK429286A	0.72	0.69
QS11	0.73	0.72
LFM-A13	0.73	0.75
Cytarabine	0.73	0.70
Embelin	0.73	0.79
Bleomycin	0.73	0.82
PD173074	0.74	0.70
Lestaurtinib	0.74	0.74
Tivozanib	0.74	-0.30
SGC0946	0.75	0.72
VNLG/124	0.75	0.81
Bicalutamide	0.75	0.70
BX795	0.75	0.68
CHIR-99021	0.76	0.78

**Wee1 Inhibitor**	0.78	0.79
**JNK Inhibitor VIII**	0.78	0.79
**Selisistat**	0.80	0.13
**GW-2580**	0.80	0.81
**Midostaurin**	0.81	0.81
**Doramapimod**	0.81	0.78
**HG6-64-1**	0.83	0.87
**PF-562271**	0.83	0.82
**Thapsigargin**	0.88	0.90
**Gefitinib**	0.90	0.89
**Olaparib**	0.91	0.87
**Talazoparib**	0.96	0.95
