# Supplementary material for: Genome-wide cross-cancer analysis illustrates the critical role of bimodal miRNA in patient survival and drug responses to PI3K inhibitors
Source: PLoS Comput Biol. 2022 May 31;18(5):e1010109. doi: 10.1371/journal.pcbi.1010109 (PMC9187341; doi:10.1371/journal.pcbi.1010109)
Supplement: S4 Table — qPCR amplification efficiency is calculated based on the slope of the standard curve. Slopes between -3.30 ± 0.20 and amplification efficiencies of 100 ± 10% are typically considered acceptable. (PDF) [file pcbi.1010109.s004.pdf]

Table S4. mRNA primers. qPCR amplification efficiency is calculated based on the slope of the standard curve. Slopes between -3.30 ± 0.20 and amplification efficiencies of 100 ± 10% are typically considered acceptable.

Ensembl ID	Common Name	Position	Primers (5' → 3')	Efficiency
ENST00000616317.5	Acetyl-CoA carboxylase alpha	Forward +681	AGGATGAGATCAGCAACCTGGTGA	108.80%
		Reverse +763	ATCTGAGCCAACAGAAGCAGGTGA	
ENST00000349310.7	AKT serine/threonine kinase 1	Forward +446	TATTGTGAAGGAGGGTTGGCTG	102.15%
		Reverse +525	CATTCTTGAGGAGGAAGTAGCGTG	
ENST00000392038.6	AKT serine/threonine kinase 2	Forward +341	GCGTGGTGAATACATCAAGACCTG	103.33%
		Reverse +415	TTGTACCCAATGAAGGAGCCGT	
ENST00000263826.9	AKT serine/threonine kinase 3	Forward +387	CAGAGGAAAGGGAAGAATGGACAG	102.42%
		Reverse +462	ATTCTCTCCTCTTCTTGCCTCTGC	
ENST00000404338.7	Rho GTPase activating protein 35	Forward +3615	TCCATACGAAACAGACGAAGACC	93.22%
		Reverse +3693	GGGCTTTGGTTTCTTAGTGTTCC	
ENST00000324774.9	Calcium/calmodulin dependent protein kinase kinase 2	Forward +2376	TGCTCACCAAAAAACCAACCAGGG	100.92%
		Reverse +2446	TTGTCTTCGCTGCCTTGCTTCCTT	
ENST00000261254.7	Cyclin D2	Forward +644	TTACACCGACAACTCCATCAAGCC	107.98%
		Reverse +718	TTCCACTTCAACTTCCCCAGCA	
ENST00000257904.10	Cyclin dependent kinase 4	Forward +512	TTCCCATCAGCACAGTTCGTGA	114.90%
		Reverse +576	ATTGGGATGCTCAAAAGCCTCC	
ENST00000424848.2	Cyclin dependent kinase 6	Forward +114	TCAGGTGCAATGATTCTGG	93.57%
		Reverse +182	GTCCTCAACACAGACACGATT	
ENST00000265641.10	Carnitine palmitoyltransferase 1A	Forward +290	ATTCAAGAACGGCATCATCACTGG	96.16%
		Reverse +384	TCTTGGCGTACATCGTTGTCATCA	
ENST00000357727.6	cAMP responsive element binding protein 5	Forward +1748	ATCTAAGTCCAGAGAGTAGC	99.68%
		Reverse +1829	GTGATGGTATTATGCTGG	
ENST00000300574.2	CRK proto-oncogene	Forward +886	AATGCCTACGACAAGACAGCCTT	99.12%
		Reverse +995	TGACCTCGTTTGCCATTACACTC	
ENST00000263026.9	Eukaryotic elongation factor 2 kinase	Forward +1872	GAAGTACGAGTCTGACGA	106.52%
		Reverse +1949	GAGGAGTTGAGGAGATTC	
ENST00000295956.8	Filamin B	Forward +1270	AACATCGCCAATAAGCCCACCTAC	90.56%
		Reverse +1349	TCTTCCACCTCCACACCAATGTCA	
ENST00000354785.9	Fibronectin 1	Forward +768	TAAGCTGTACCATCGCAAACCGC	97.23%
		Reverse +848	TCTCATGTGGTCTCCTCCAGGTGT	
ENST00000406360.2	Forkhead box O3	Forward +1327	GGCAAGCACAGAGTTGGATGAAGT	99.93%
		Reverse +1427	ACGGCTTGCTTACTGAAGGTGACA	
ENST00000337514.10	Insulin like growth factor 1 receptor	Forward +337	CACACCATGTCCTCCTCGCA	100.10%
		Reverse +391	TGAAGGTGAGCAGGCACAGC	
ENST00000305123.5	Insulin receptor substrate 1	Forward +3694	GAGCCCGGGGGAATATGTCAA	109.10%
		Reverse +3763	AGCCACCGGGCCAGACAA	
ENST00000311936.7	KRAS proto-oncogene, GTPase	Forward +360	CGACACAGCAGGTCAAGAGGAG	99.68%
		Reverse +426	AAAGCCCTCCCCAGTCCTCA	
ENST00000311936.7	Ribosomal protein L7a	Forward +145	TTTGGCATTGGACAGGACATCC	105.10%
		Reverse +208	AGCGGGGCCATTTCACAAAG	
ENST00000215832.10	Mitogen-activated protein kinase 1	Forward +1117	CACCCATATCTGGAGCAGTATTACG	99.96%
		Reverse +1216	GCTTTTCCTTAGGCAAGTCATCC	
ENST00000369026.2	MCL1, BCL2 family	Forward +993	CTGGGATGGGTTTGTGGAGTTCTT	97.86%

ENST00000369020.2	apoptosis regulator	Reverse +1064	AAAGCCAGCAGCACATTCCTGA	[illegible]
ENST00000369535.4	NRAS proto-oncogene, GTPase	Forward +231	GCTTGAGGTTCTTGCTGGTGTGAA	101.96%
		Reverse +287	TGCTCCAACCACCACCAGTTTGTA	
ENST00000342085.8	3-phosphoinositide dependent protein kinase 1	Forward +704	CACGGCTGAGATTGTGTCTGCTTTA	90.54%
		Reverse +773	CGGTTTAAGGTCCCTGTGAATGATG	
ENST00000367080.7	6-phosphofructo-2-kinase/fructose-2,6-bisphosphatase 2	Forward +92	TAGGAGAGATCGTAGGACATC	108.03%
		Reverse +152	TCTGTTCTGAGGAAGATGC	
ENST00000232375.7	6-phosphofructo-2-kinase/fructose-2,6-bisphosphatase 4	Forward +220	CCAACTGCCCAACTCTCATT	91.66%
		Reverse +338	AACATTGAACTCCCGAGTGG	
ENST00000265970.11	Phosphatidylinositol-4-phosphate 3-kinase catalytic subunit type 2 alpha	Forward +3516	CAAATGTCTCTCAACTGGC	109.64%
		Reverse +3589	GGATTTTCCTGAGGGTATC	
ENST00000367187.7	Phosphatidylinositol-4-phosphate 3-kinase catalytic subunit type 2 beta	Forward +440	AACTACAGTTTGCCTTGCCAGG	97.05%
		Reverse +510	GTCAGTTCTGGAGGGTTGTGACAT	
ENST00000262039.8	Phosphatidylinositol 3-kinase catalytic subunit type 3	Forward +1142	GGGAAAATGGAAGCCGATGGAT	98.80%
		Reverse +1229	AACAGCATAACGCCTCACAGTTGG	
ENST00000263967.3	Phosphatidylinositol-4,5-bisphosphate 3-kinase catalytic subunit alpha	Forward +2374	GAGGCGACCAGATTTCAT	98.48%
		Reverse +2457	TCAAGCCTGAGGTTTCCT	
ENST00000289153.6	Phosphatidylinositol-4,5-bisphosphate 3-kinase catalytic subunit beta	Forward +2088	TTGGTGTCATCCTTGAAGC	100.73%
		Reverse +2165	TGAGTGCTTCAACCTGCTT	
ENST00000377346.8	Phosphatidylinositol-4,5-bisphosphate 3-kinase catalytic subunit delta	Forward +528	GCTCATCAACTCACAGATCAGCCT	89.82%
		Reverse +593	TCGCACAAGGAGTCAAACTCGT	
ENST00000320694.12	Phosphoinositide-3-kinase regulatory subunit 1	Forward +1383	CGAGGGAAGCGAGATGGCA	103.16%
		Reverse +1462	CCGTCCACCACTACAGAGCAGG	
ENST00000222254.12	Phosphoinositide-3-kinase regulatory subunit 2	Forward +1061	GTACAGACTGGTCCCTGAGCGAC	93.09%
		Reverse +1125	CTTAATGCCGTCAGCCAGGG	
ENST00000262741.9	Phosphoinositide-3-kinase regulatory subunit 3	Forward +849	GAAGGACAGTTCTGTTTCTCT	106.86%
		Reverse +914	ACCTCCTCCCTTGAAATAT	
ENST00000311129.9	Protein phosphatase 2 scaffold subunit A beta	Forward +311	TTGCCCACTGTCTGCTGCCT	108.28%
		Reverse +390	GGACTCCACAGCCTTGTCACGA	
ENST00000371244.8	Protein kinase AMP-activated catalytic subunit alpha 2	Forward +389	ATGGACGGGTTGAAGAGAT	98.44%
		Reverse +455	CAGTAATCCACAGCAGACAGA	
ENST00000254101.3	Protein kinase AMP-activated non-catalytic subunit beta 2	Forward +242	ATCATGGTGGGGAGTACGGACGACC	109.17%
		Reverse +311	CTTTGTCCCCAGGGAGCTTGGAGTC	
ENST00000413366.7	Protein kinase C alpha	Forward +412	GGATGAAATGTGACACCTGCG	92.31%
		Reverse +477	AGAGGCTGGGGACATTGATGA	
ENST00000355622.8	Toll like receptor 4	Forward +1447	CAGAGTTGCTTTCAATGGCATC	110.23%
		Reverse +1728	AGACTGTAATCAAGAACCTGGAGG	
ENST00000371199.7	X-linked inhibitor of apoptosis	Forward +1204	ACTGTGGAGGAGGGCTAACTGATT	96.49%
		Reverse +1280	GCACCCTGGATACCATTTAGCA	
ENST00000264335.12	Tyrosine 3-monooxygenase/tryptophan 5-monooxygenase activation	Forward +318	CTGAGCGATACGACGAAATGGT	105.55%
		Reverse +375	TCCACATCCATCCCTGCTACTT	
ENST00000307630.4	Tyrosine 3-monooxygenase/tryptophan 5-monooxygenase activation	Forward +397	TTCCTGGAGGGTCATCAGTAGCAT	99.09%
		Reverse +474	GCACGGACCATCTCAATCTTCTTC	
