# Supplementary material for: Genome-wide cross-cancer analysis illustrates the critical role of bimodal miRNA in patient survival and drug responses to PI3K inhibitors
Source: PLoS Comput Biol. 2022 May 31;18(5):e1010109. doi: 10.1371/journal.pcbi.1010109 (PMC9187341; doi:10.1371/journal.pcbi.1010109)
Supplement: S5 Table — All primers are Taqman Advanced miRNA Assay. (PDF) [file pcbi.1010109.s005.pdf]

Table S5. miRNA probes. All primers are Taqman Advanced miRNA Assay.

miRNA	Assay Number	Mature Sequence
hsa-miR-105-5p	477865_mir	UCAAAUGCUCAGACUCCUGUGGU
hsa-miR-767-5p	479176_mir	UGCACCAUGGUUGUCUGAGCAUG
hsa-miR-9-5p	478214_mir	UCUUUGGUUAUCUAGCUGUAUGA
U6 snRNA	1973	GTGCTCGCTTCGGCAGCACATATACTAAA ATTGGAACGATACAGAGAAGATTAGCATG GCCCCTGCGCAAGGATGACACGCAAATTC GTGAAGCGTTCCATATTTT
