# Supplementary material for: Genome-wide cross-cancer analysis illustrates the critical role of bimodal miRNA in patient survival and drug responses to PI3K inhibitors
Source: PLoS Comput Biol. 2022 May 31;18(5):e1010109. doi: 10.1371/journal.pcbi.1010109 (PMC9187341; doi:10.1371/journal.pcbi.1010109)
Supplement: S6 Table — IF: immunofluorescence, WB: western blot, RIP: RNA immunoprecipitation. (PDF) [file pcbi.1010109.s006.pdf]

**Table S6. Antibody information.** IF: immunofluorescence, WB: western blot, RIP: RNA immunoprecipitation.

| Antibody | Company | Cat No | Use |
|---|---|---|---|
| Ki-67 | BioLegend | 151202 | IF |
| Hoechst 33342 | Invitrogen | H1399 | IF |
| Actin | Abcam | sc-1616-R | WB |
| AKT | Cell Signaling | 9272 | WB |
| ERK | Cell Signaling | 4695S | WB |
| FOXO3 | Cell Signaling | 12829S | WB |
| HRP-IgG | Cell Signaling | 7074S | WB |
| IRS1 | Cell Signaling | 2390S | WB |
| KRAS | Cell Signaling | 8955S | WB |
| p-AKT | Cell Signaling | 9271S | WB |
| PDK1 | Cell Signaling | 5662S | WB |
| p-ERK | Cell Signaling | 4370S | WB |
| p-FOXO3 | Cell Signaling | 9466S | WB |
| p-IRS1 | Cell Signaling | 2491S | WB |
| AGO2 | Sigma | SAB4200085 | RIP |
| Rat IgG | Sigma | I4131 | RIP |
| Rat IgG (produced in rabbit) | Sigma | R9255 | RIP |
