# Supplementary figures and images for: Genome-wide cross-cancer analysis illustrates the critical role of bimodal miRNA in patient survival and drug responses to PI3K inhibitors
Source: PLoS Comput Biol. 2022 May 31;18(5):e1010109. doi: 10.1371/journal.pcbi.1010109 (PMC9187341; doi:10.1371/journal.pcbi.1010109)

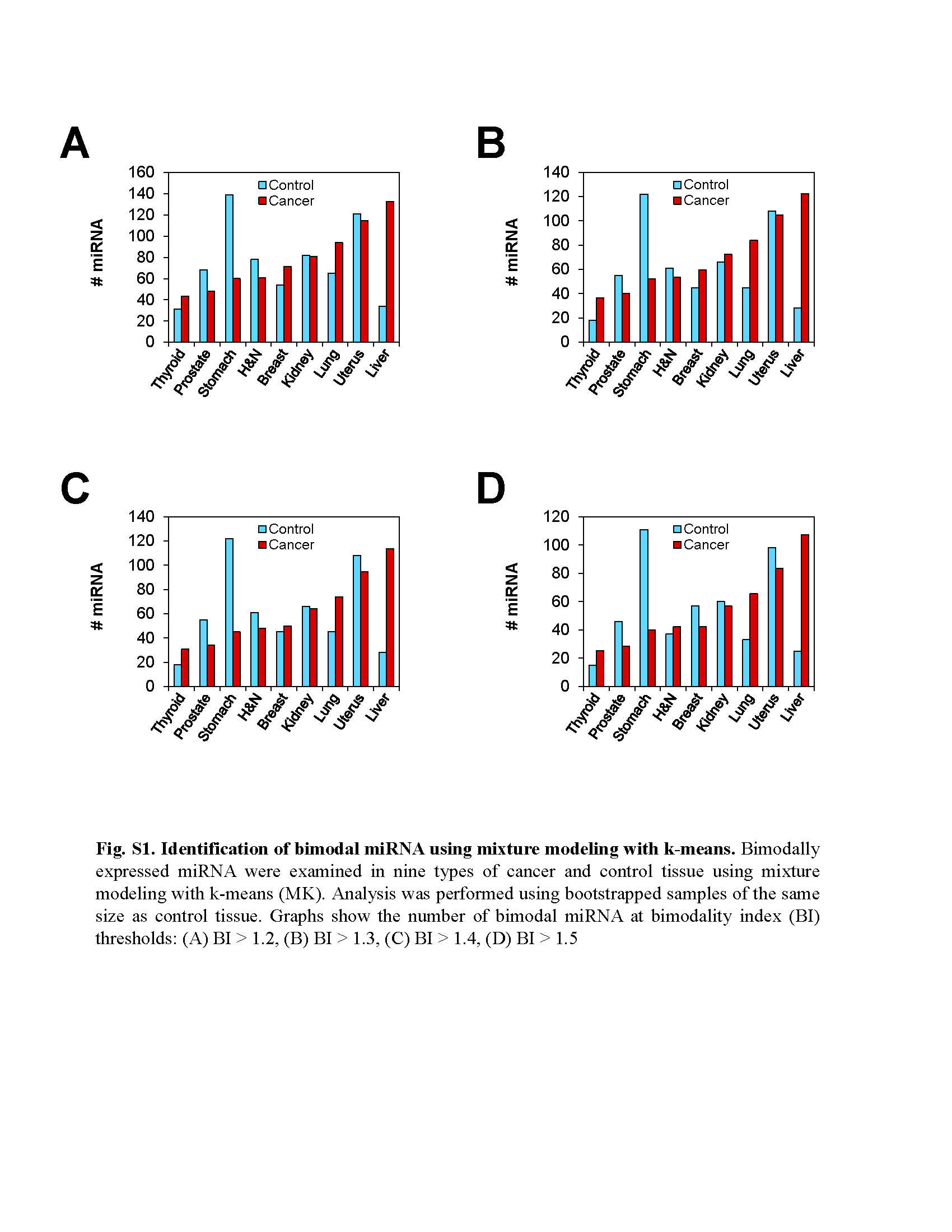

Supplement: S1 Fig — Bimodally expressed miRNA were examined in nine types of cancer and control tissue using mixture modeling with k-means (MK). Analysis was performed using bootstrapped samples of the same size as control tissue. Graphs show the number of bimodal miRNA at bimodality index (BI) thresholds: (A) BI > 1.2, (B) BI > 1.3, (C) BI > 1.4, (D) BI > 1.5. (TIFF) [file pcbi.1010109.s007.tiff]

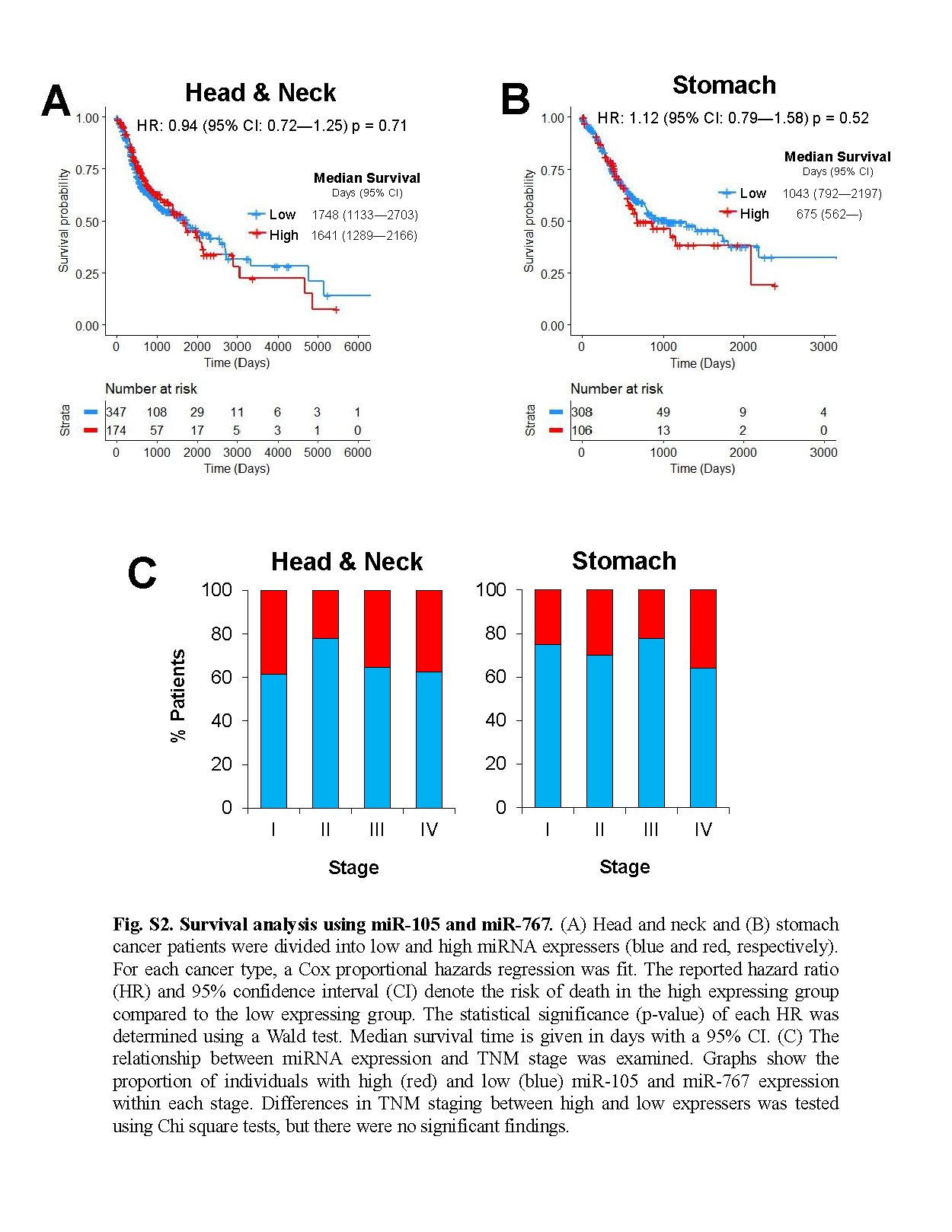

Supplement: S2 Fig — (A) Head and neck and (B) stomach cancer patients were divided into low and high miRNA expressers (blue and red, respectively). For each cancer type, a Cox proportional hazards regression was fit. The reported hazard ratio (HR) and 95% confidence interval (CI) denote the risk of death in the high expressing group compared to the low expressing group. The statistical significance (p-value) of each HR was determined using a Wald test. Median survival time is given in days with a 95% CI. (C) The relationship between miRNA expression and TNM stage was examined. Graphs show the proportion of individuals with high (red) and low (blue) miR-105 and miR-767 expression within each stage. Differences in TNM staging between high and low expressers was tested using Chi square tests, but there were no significant findings. (TIFF) [file pcbi.1010109.s008.tiff]

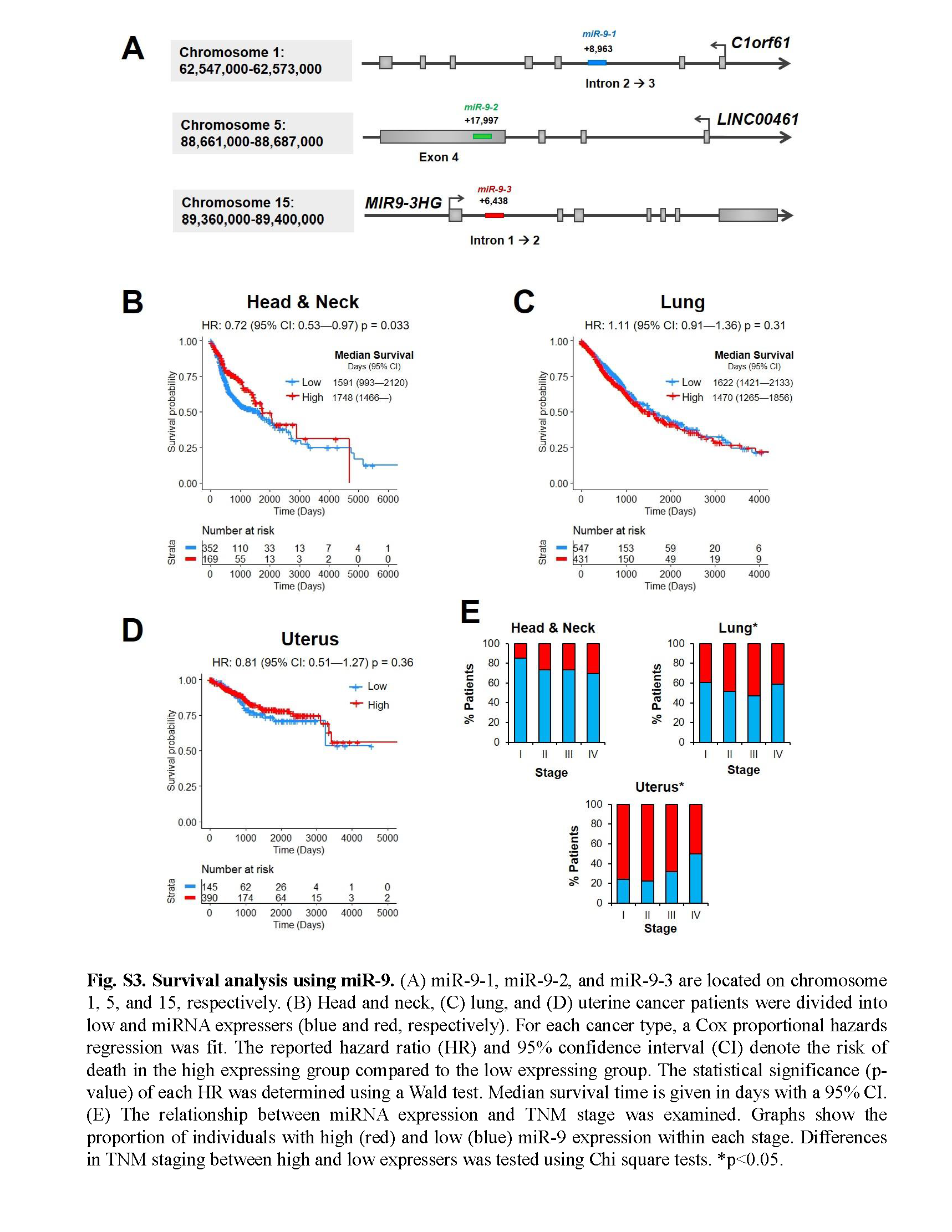

Supplement: S3 Fig — (A) miR-9-1, miR-9-2, and miR-9-3 are located on chromosome 1, 5, and 15, respectively. (B) Head and neck, (C) lung, and (D) uterine cancer patients were divided into low and miRNA expressers (blue and red, respectively). For each cancer type, a Cox proportional hazards regression was fit. The reported hazard ratio (HR) and 95% confidence interval (CI) denote the risk of death in the high expressing group compared to the low expressing group. The statistical significance (pvalue) of each HR was determined using a Wald test. Median survival time is given in days with a 95% CI. (E) The relationship between miRNA expression and TNM stage was examined. Graphs show the proportion of individuals with high (red) and low (blue) miR-9 expression within each stage. Differences in TNM staging between high and low expressers was tested using Chi square tests. *p<0.05. (TIFF) [file pcbi.1010109.s009.tiff]

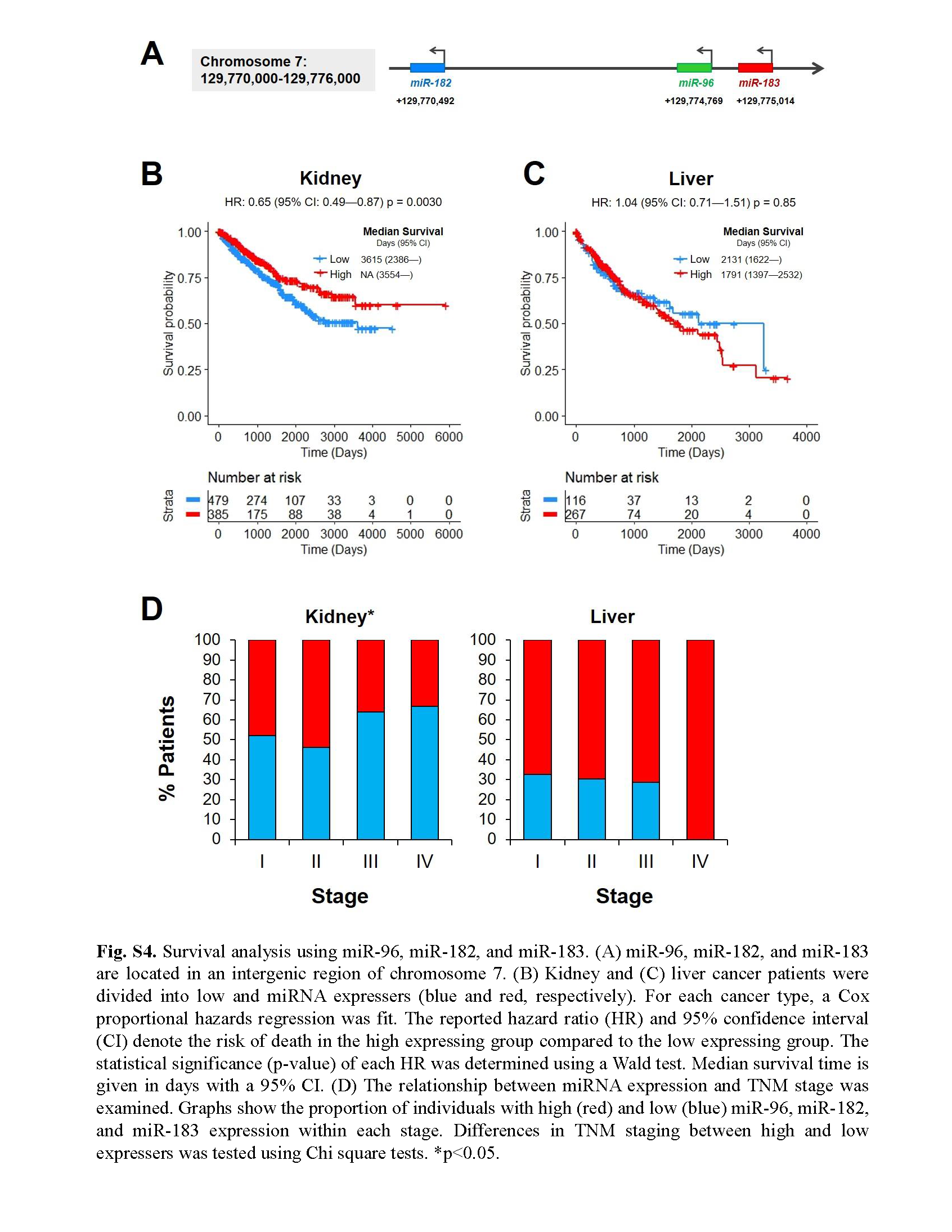

Supplement: S4 Fig — (A) miR-96, miR-182, and miR-183 are located in an intergenic region of chromosome 7. (B) Kidney and (C) liver cancer patients were divided into low and miRNA expressers (blue and red, respectively). For each cancer type, a Cox proportional hazards regression was fit. The reported hazard ratio (HR) and 95% confidence interval (CI) denote the risk of death in the high expressing group compared to the low expressing group. The statistical significance (p-value) of each HR was determined using a Wald test. Median survival time is given in days with a 95% CI. (D) The relationship between miRNA expression and TNM stage was examined. Graphs show the proportion of individuals with high (red) and low (blue) miR-96, miR-182, and miR-183 expression within each stage. Differences in TNM staging between high and low expressers was tested using Chi square tests. *p<0.05. (TIFF) [file pcbi.1010109.s010.tiff]

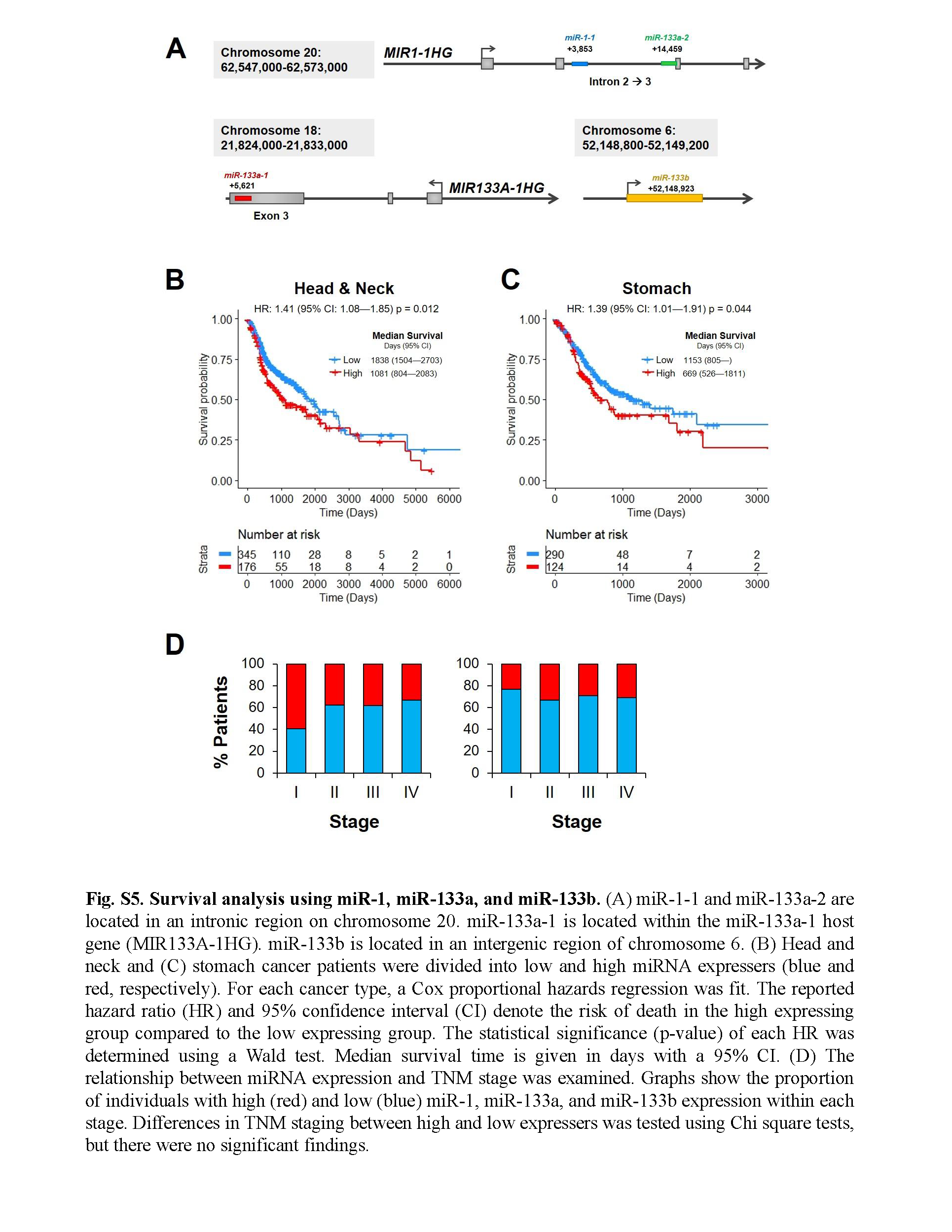

Supplement: S5 Fig — (A) miR-1-1 and miR-133a-2 are located in an intronic region on chromosome 20. miR-133a-1 is located within the miR-133a-1 host gene (MIR133A-1HG). miR-133b is located in an intergenic region of chromosome 6. (B) Head and neck and (C) stomach cancer patients were divided into low and high miRNA expressers (blue and red, respectively). For each cancer type, a Cox proportional hazards regression was fit. The reported hazard ratio (HR) and 95% confidence interval (CI) denote the risk of death in the high expressing group compared to the low expressing group. The statistical significance (p-value) of each HR was determined using a Wald test. Median survival time is given in days with a 95% CI. (D) The relationship between miRNA expression and TNM stage was examined. Graphs show the proportion of individuals with high (red) and low (blue) miR-1, miR-133a, and miR-133b expression within each stage. Differences in TNM staging between high and low expressers was tested using Chi square tests, but there were no significant findings. (TIFF) [file pcbi.1010109.s011.tiff]

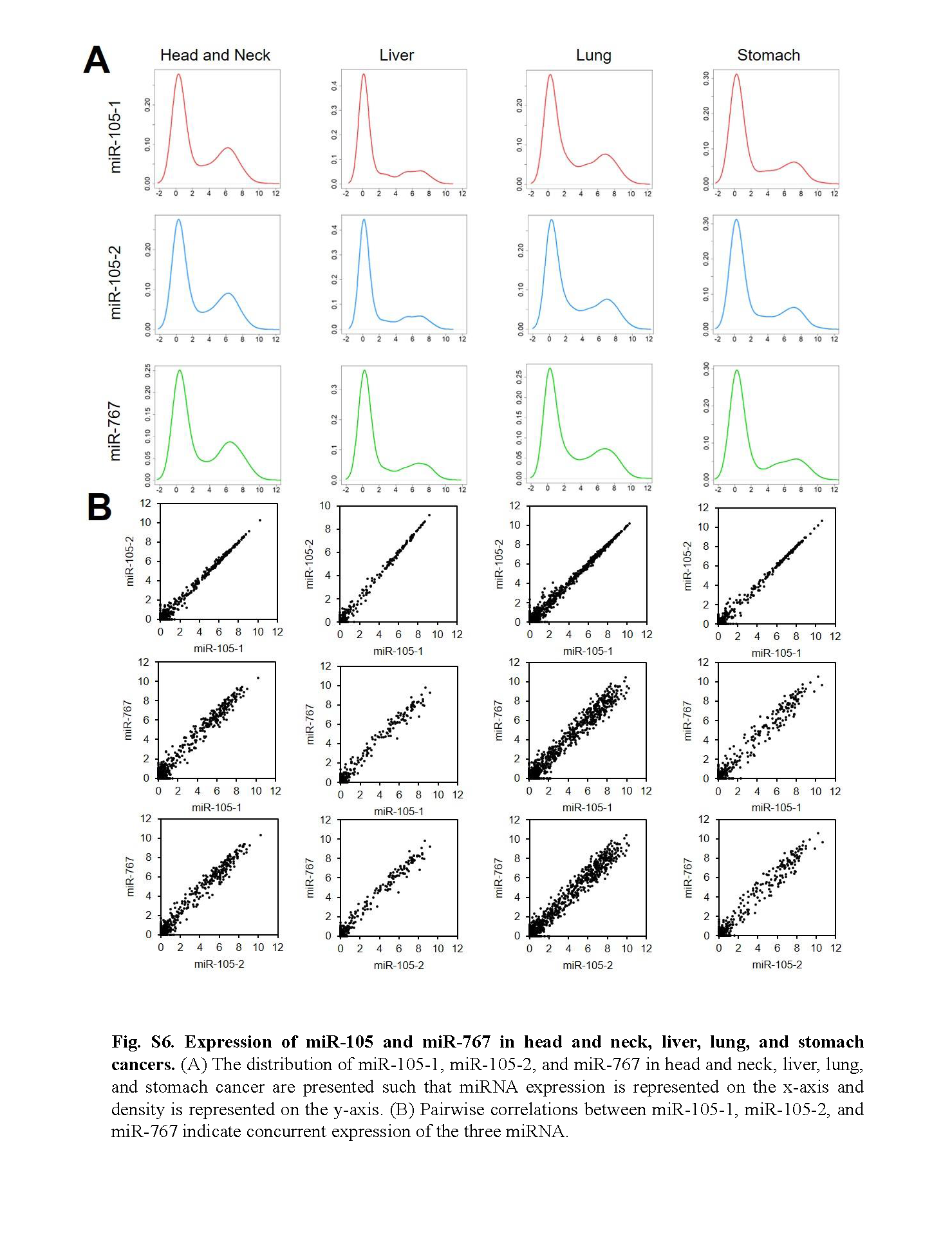

Supplement: S6 Fig — (A) The distribution of miR-105-1, miR-105-2, and miR-767 in head and neck, liver, lung, and stomach cancer are presented such that miRNA expression is represented on the x-axis and density is represented on the y-axis. (B) Pairwise correlations between miR-105-1, miR-105-2, and miR-767 indicate concurrent expression of the three miRNA. (TIFF) [file pcbi.1010109.s012.tiff]

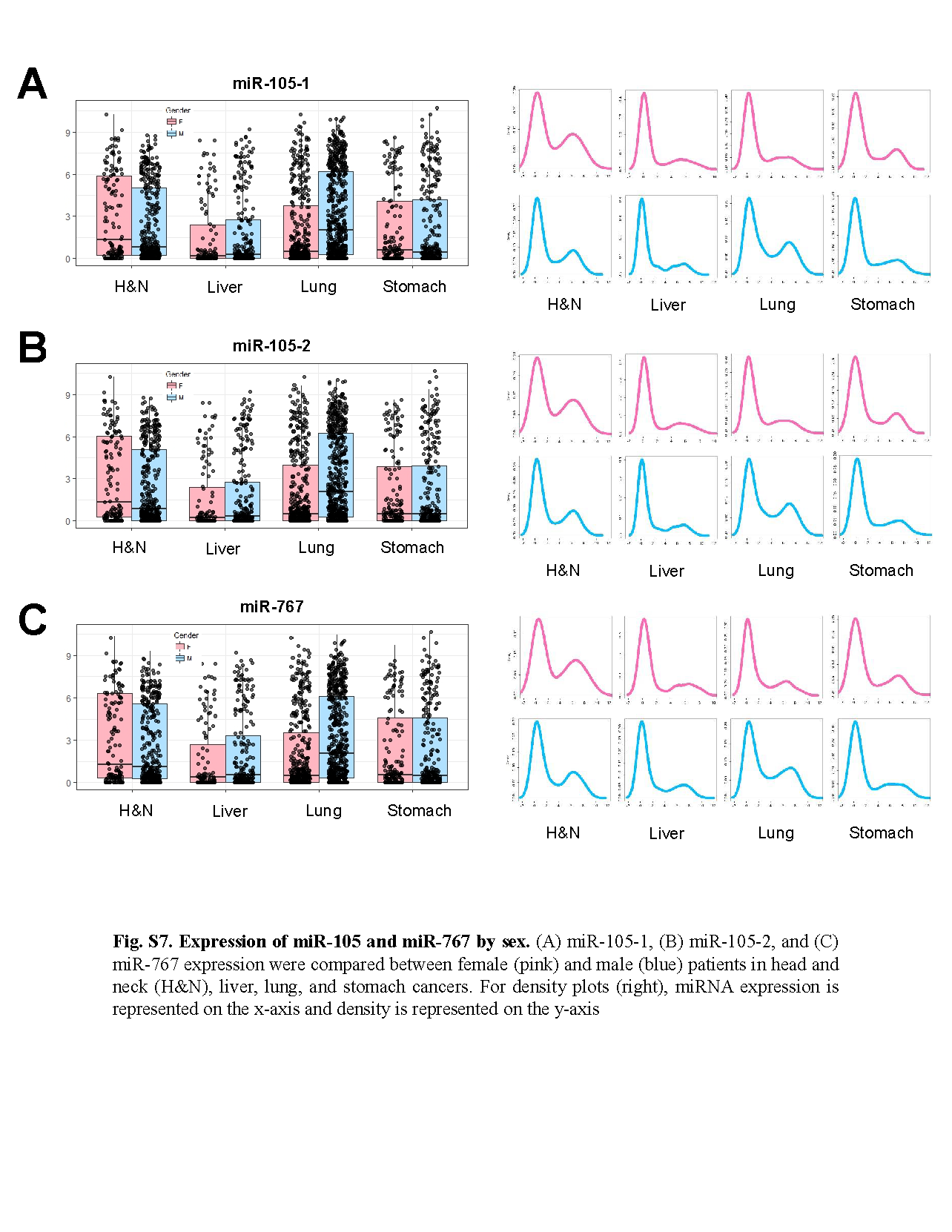

Supplement: S7 Fig — (A) miR-105-1, (B) miR-105-2, and (C) miR-767 expression were compared between female (pink) and male (blue) patients in head and neck (H&N), liver, lung, and stomach cancers. For density plots (right), miRNA expression is represented on the x-axis and density is represented on the y-axis. (TIFF) [file pcbi.1010109.s013.tiff]

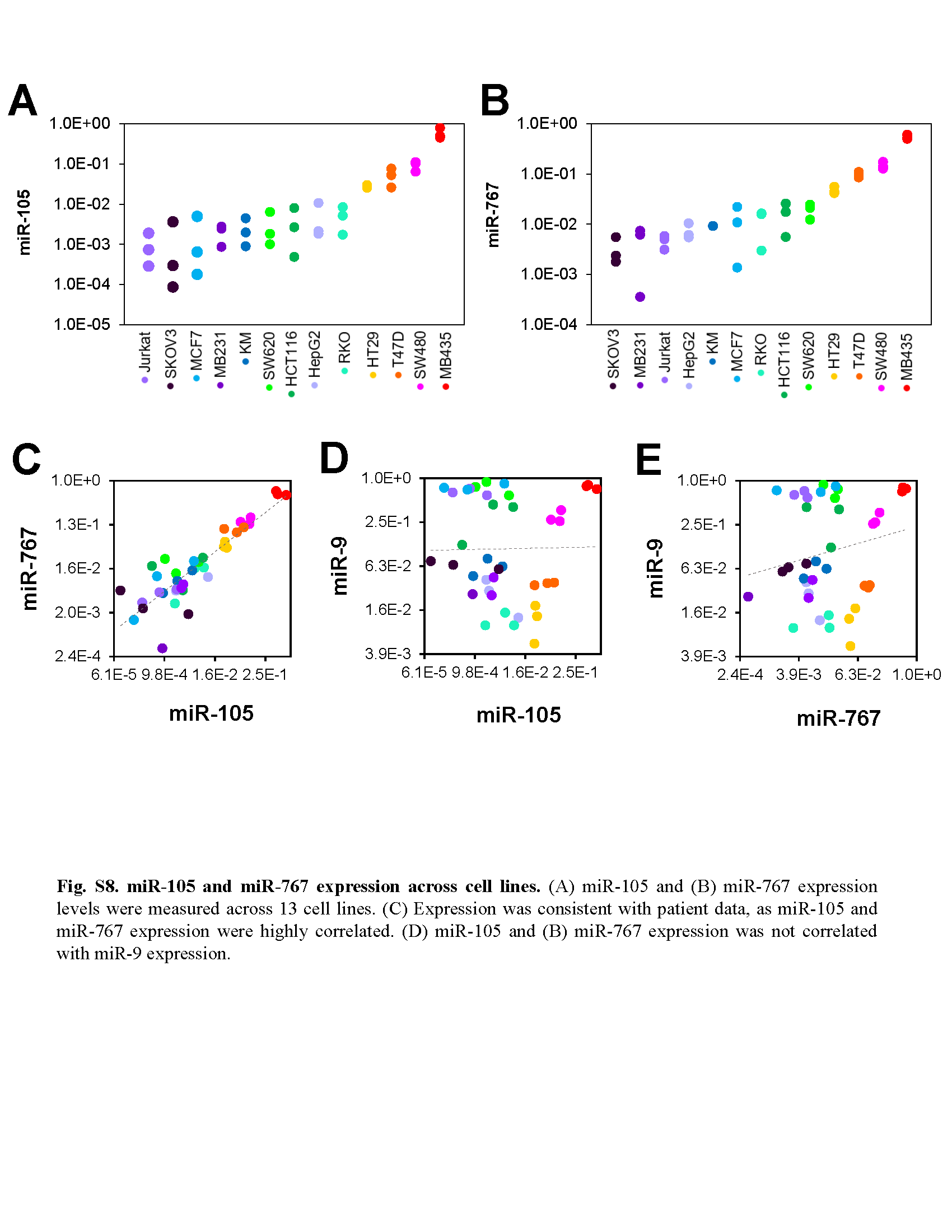

Supplement: S8 Fig — (A) miR-105 and (B) miR-767 expression levels were measured across 13 cell lines. (C) Expression was consistent with patient data, as miR-105 and miR-767 expression were highly correlated. (D) miR-105 and (B) miR-767 expression was not correlated with miR-9 expression. (TIFF) [file pcbi.1010109.s014.tiff]

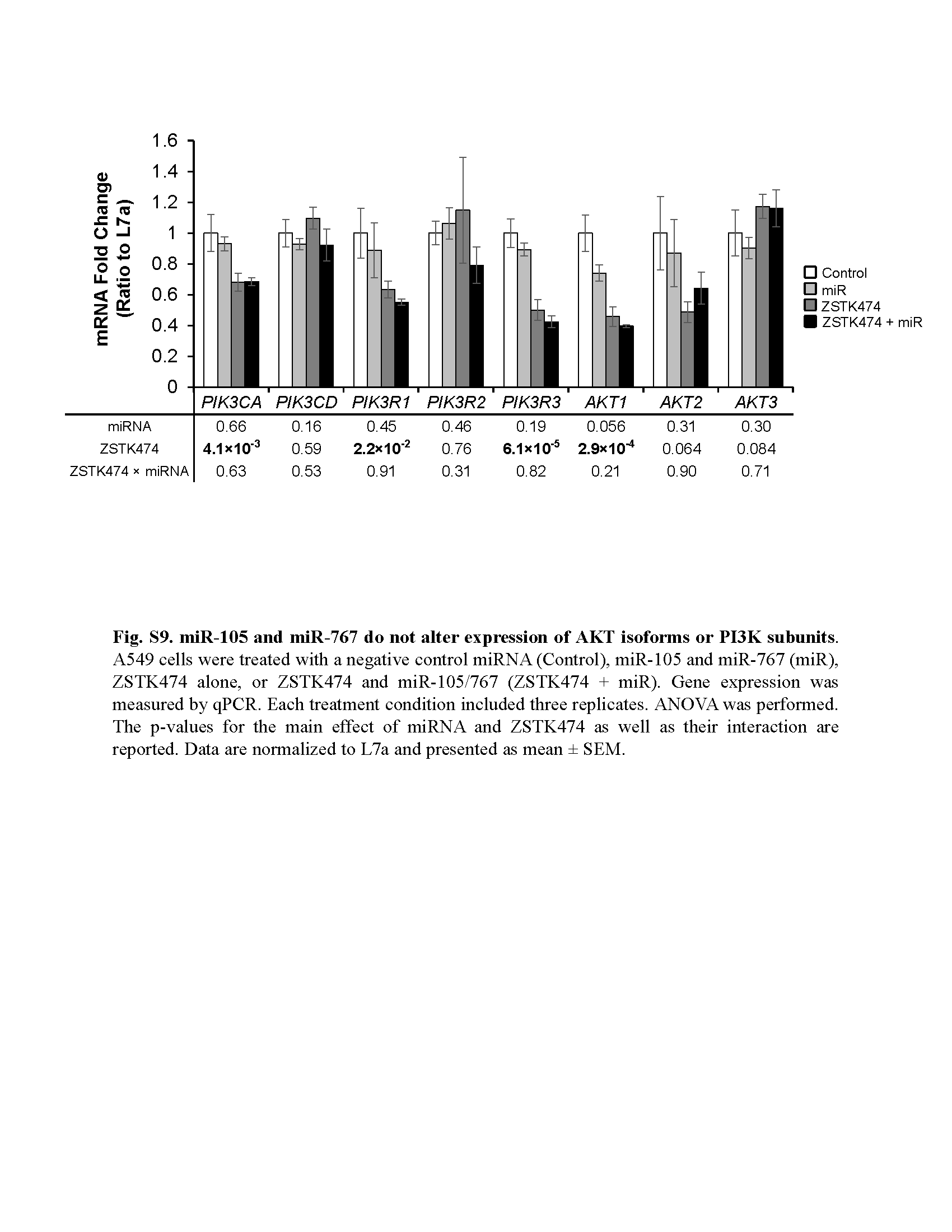

Supplement: S9 Fig — A549 cells were treated with a negative control miRNA (Control), miR-105 and miR-767 (miR), ZSTK474 alone, or ZSTK474 and miR-105/767 (ZSTK474 + miR). Gene expression was measured by qPCR. Each treatment condition included three replicates. ANOVA was performed. The p-values for the main effect of miRNA and ZSTK474 as well as their interaction are reported. Data are normalized to L7a and presented as mean ± SEM. (TIFF) [file pcbi.1010109.s015.tiff]
