# Supplementary material for: Genome-wide cross-cancer analysis illustrates the critical role of bimodal miRNA in patient survival and drug responses to PI3K inhibitors
Source: PLoS Comput Biol. 2022 May 31;18(5):e1010109. doi: 10.1371/journal.pcbi.1010109 (PMC9187341; doi:10.1371/journal.pcbi.1010109)
Supplement: S1 Supplementary Methods — (DOCX) [file pcbi.1010109.s016.docx]

**Supplementary Methods**

Cell lines and treatments

Several cell lines were utilized for miRNA quantification, including HCT116, HepG2, HT29, Jurkat, KM, MB231, MB435, MCF7, RKO, SKOV3, SW480, SW620, and T47D. For drug sensitivity studies, A549 and HepG2 cells were used. Cells were purchased from ATCC (Manassas, VA) between 2008 and 2018. Cells were cultured according to a standard procedure in our laboratory [1].

miRNA mimics and transfection reagents were purchased from Dharmacon (Fisher Scientific) and overexpression was performed according to the manufacturer’s instructions. Briefly, cells were seeded and incubated overnight before transfection. A final concentration of 10 nM of miR-105 and miR-767 mimics (5 nM each) was mixed with Dharmafect Duo transfection reagent (Dharmacon) and incubated with target cells. Cells were then incubated for 24 hours. As a negative control, 10 nM of a scrambled miRNA sequence based on C. elegans that has minimal sequence identity in human, mouse, and rat was used. For all experiments using ZSTK474 treatment, cells were first treated with either miR-105 and miR-767 mimics or negative control for 24 hours before ZSTK474 treatment was administered.

miRNA and mRNA expression

Cells were directly lysed in TRI Reagent (Zymo Research, Irvine, CA). Total RNA was extracted using Direct-zol RNA MiniPrep Plus (Zymo Research) with in-column DNase I treatment to eliminate genomic DNA contamination. RNA concentration was measured by the NanoDrop Spectrophotometer 2000 (Thermo Fisher Scientific, Waltham, MA).

For mRNA expression, cDNA synthesis was performed using the High Capacity cDNA Reverse Transcription Kit (Applied Biosystems, Foster City, CA) with amplification using the 2720 Thermal Cycler (Applied Biosystems). After synthesis, a serially diluted standard curve and cDNA samples were analyzed using quantitative real-time PCR (qPCR). Gene expression was assessed with PowerUp SYBR Green PCR Master Mix (Applied Biosystems) using the StepOne Real-Time PCR System (Applied Biosystems). The reaction was performed in 40 cycles and standard curves with slope of -3.30 ± 0.20 and amplification efficiencies of 100 ± 10% were accepted. All mRNA primers were designed using Vector NTI software (Invitrogen Corporation, Carlsbad, CA) and synthesized by Integrated DNA Technologies (IDT, Newark, NJ). Primers are described in **S4 Table**. Gene expression was normalized to the expression of the internal control gene ribosomal protein L7a (*L7a*).

For miRNA expression, 10 ng total RNA was used for cDNA synthesis using the TaqMan miRNA Reverse Transcription Kit. cDNA was diluted and qPCR was performed using TaqMan Fast Advanced Master Mix (Applied Biosystems) and TaqMan miRNA Assay primers that were specific to miR-105 or miR-767 (Applied Biosystems). The reaction was performed using a StepOne Real-Time PCR System and miRNA were quantified via the ΔΔCT method. A TaqMan miRNA Control Assay for U6 snRNA was included for normalization. miRNA primers can be found in **S5 Table**.

Immunofluorescence

Cells grown on coverslips were washed with PBS, fixed in 4% PFA, permeabilized with 0.5% Triton X-100 (diluted in PBS), and blocked with blocking buffer (5% normal goat serum, 0.1% Triton X-100 diluted in PBS). Cells were incubated with Alexa Fluor® 488 anti-mouse/human Ki-67 primary antibody (1:200 dilution, BioLegend, San Diego, CA; **S6 Table**) then incubated with Hoechst 33342 (1:200 dilution, Invitrogen; **S6 Table**). Cells were mounted with Prolong-Gold Antifade Reagent (Molecular Probes by Life Technologies, Carlsbad, CA). Pictures were taken using the Confocal LSM 700 microscope (Carl Zeiss Microscopy, LLC, United States) with Zen software (Carl Zeiss AG) at magnification of 63X. To quantify expression, three 67.74 µm x 67.74 µm area were randomly selected in each group, and analysis was performed using ImageJ software.

Cell viability assay

Cell viability was determined by WST-1 assay (Premix WST-1 Cell Proliferation Assay System, Takara Bio, Mountain View, CA). Cells were incubated in WST-1 for 2.5 hours. Absorbance was measured at 450 nm using the Synergy H1 Microplate Reader (Biotek, Winooski, VT). For each sample, background absorbance at 650 nm was subtracted.

Flow cytometry

For cell cycle analysis, cells were trypsinized, washed with PBS, and resuspended in 70% ethanol. Cells were stored at 4 °C and on the day of analysis, cells were pelleted and decanted. The cells were washed in PBS and resuspended in propidium iodide (PI) staining solution (100 ml 0.1% (v/v) Triton X-100 in PBS, 20 mg DNase-free RNase A, 2 mg PI). Cells were incubated at room temperature for 30 min. Flow cytometry was performed using the BD LSR II Flow Cytometry Analyzer (BD Biosciences, San Jose, CA).

For apoptosis analysis, live cells were washed with PBS and resuspended in binding buffer (10 mM HEPES-NaOH, pH 7.4, 140 mM NaCl, 5 mM CaCl_2_). For each sample, 1 μg/ml fluorescein-conjugated annexin V (FITC Annexin V, BioLegend, San Diego, CA) was added and incubated in the dark for 5 min at room temperature. PI (Sigma-Aldrich, St. Louis, MO) was then added for a final concentration of 1.0 μg/ml. The reaction was incubated in the dark at room temperature for 5 min. Cells were analyzed using the BD LSR II Flow Cytometry Analyzer. Green annexin V fluorescence was collected at 530 ± 20 nm and red PI fluorescence was collected above 600 nm. Compensation was set based on cells stained with annexin V only and with PI only. Unstained cells were considered non-apoptotic, annexin V positive/PI negative cells were considered early apoptotic, and annexin V positive/PI positive cells were considered late apoptotic. Analysis was performed in FCS Express version 5.

Western blot analysis

For each treatment, cells were lysed in protein lysis buffer (0.125 M Tris HCl, pH 6.8), 1% SDS, 0.04% bromophenol blue, 20% glycerol, 5% 2-mercaptoethanol, 1x proteinase inhibitor, 1x phosphatase inhibitor cocktail 2 and 3) and sonicated using the Fisher Scientific model 100 Sonic Dismembrator. For gel electrophoresis, protein samples were boiled and 20 μg of sample was loaded into 1 mm 12% acrylamide gels. Additionally, 10 μl of Precision Plus Protein Dual Color Standard (Bio-Rad Laboratories, Hercules, CA) was used.

Gels were transferred to PVDF membranes using the Trans-Blot Turbo system (Bio-Rad Laboratories). Membranes were blocked in 3% (w/v) BSA in 1x TBS/0.1% Tween20 (TBST) for 1 hour at room temperature. For primary incubation, antibodies were diluted 1:1000 in 3% BSA. For phosphorylated proteins, incubation occurred at 4 ^o^C overnight. For all other proteins, incubation occurred at room temperature for 2.5 hours. Membranes were then washed in 1% (w/v) BSA in TBST. Secondary incubation with HRP-linked anti-rabbit IgG (1:5000) in 3% BSA was performed for 1 hour at room temperature. Membranes were washed in 1% BSA and incubated in Supersignal Western Blot Enhancer (Thermo Scientific). Images were acquired using the ChemiDoc XRS (Bio-Rad Laboratories), and Quantity One software (Bio-Rad Laboratories) was used to quantify signal intensity.

Membranes were stripped for 30 min at 37 ^o^C using Restore Plus Western Blot Stripping Buffer (Thermo Scientific). They were then washed twice with TBST and blocked for one hour before proceeding with primary and secondary incubation. Membranes were stripped a maximum of twice. All phosphorylated proteins were blotted on the same membrane as the corresponding total protein. For quantification, samples were run in triplicate. Total protein was normalized to beta actin and phosphorylated proteins were normalized to the corresponding total protein. All antibodies can be found in **S6 Table**.

RNA immunoprecipitation (RIP)

For each RIP, 2 million A549 cells were collected. Thus, each sample required 4 million cells, including 2 million for AGO2 and 2 million for IgG negative control. Cells were washed with PBS and incubated in nucleus swelling buffer (5mM PIPES (pH = 8.0), 85 mM KCl, 0.5% NP 40, 1x protease inhibitor, 1x phosphatase inhibitor cocktail 2 and 3) on ice. Tubes were then centrifuged and supernatant containing cell lysate was transferred to a new tube. For each sample, 10 μl was removed for input.

Dynabeads Protein G beads (Invitrogen) were bound to antibody. For each RIP, 20 μl beads were washed with 1x PBS/0.02% Tween 20 (PBST). Beads were resuspended in PBST and incubated with 2.5 μg rabbit Anti-Rat IgG antibody produced in rabbit (Sigma-Aldrich; **S6 Table**) for 1 hour at room temperature. Beads were washed and resuspended in PBST. For IgG RIP, 2.5 μg rat IgG antibody was added (**S6 Table**). For AGO2 RIP, 2.5 μg Monoclonal Anti-AGO2 antibody produced in rat (clone 11A9, Sigma-Aldrich; **S6 Table**) was used. Beads were incubated at room temperature for 1 hour then washed with PBST. Immunoprecipitation was performed by resuspending the antibody-bound beads in cell lysates and incubating at 4 ^o^C overnight. Beads were then washed in salt buffers and resuspended in PBST.

For RNA purification, TRI Reagent (Zymo Research) and chloroform were added to each RIP and input. Samples were centrifuged at 4 ^o^C and the aqueous layer was transferred to 1.5 ml tubes containing RNA purification buffer (6 μl linear acrylamide (5 mg/ml, Ambion), 60 μl 5 M ammonium acetate, 600 μl isopropanol). Samples were vortexed and precipitated at -80 ^o^C for 2 hours. Samples were washed with 70% ethanol and rehydrated in RNase-free water. Total RNA quantification, cDNA synthesis, and qPCR were performed as described above.

Referencs

1. Moody L, Xu GB, Chen H, Pan YX. Epigenetic regulation of carnitine palmitoyltransferase 1 (Cpt1a) by high fat diet. Biochim Biophys Acta Gene Regul Mech. 2019;1862(2):141-52. doi: 10.1016/j.bbagrm.2018.12.009. PubMed PMID: 30605728.
