# Supplementary material for: Genome-wide cross-cancer analysis illustrates the critical role of bimodal miRNA in patient survival and drug responses to PI3K inhibitors
Source: PLoS Comput Biol. 2022 May 31;18(5):e1010109. doi: 10.1371/journal.pcbi.1010109 (PMC9187341; doi:10.1371/journal.pcbi.1010109)
Supplement: Attachment — Submitted filename: 20211216_Plos_Reviewer_Response.docx [file pcbi.1010109.s017.docx]

Guest Editor Comments:

1. **Line 68. The authors mentioned that mixture modeling has never been used to assess genome-wide bimodal microRNA expression. This claim is too strong. I encourage them to take a look at** [**https://doi.org/10.1093/bioinformatics/btt599**](https://urldefense.com/v3/__https:/doi.org/10.1093/bioinformatics/btt599__;!!DZ3fjg!u8g7ry8wN-10Jp5R8g_tBQY64ex_rm-7DJ_PmFn442o630_Dz1NosmjGgRxQ9wew5KY$)

This statement has been edited reflect the findings of the above citation:

Additionally, only a handful of studies have applied mixture modeling to assess high-throughput sequencing data [10] and microRNA (miRNA) expression [11].

2. **Besides bimodality, trimodality seems more reasonable since there could be low, no change, and high expression for a given microRNA w.r.t. to normal samples. Please explain.**

While it is certainly possible that trimodal expression may be observed, we are specifically interested in miRNA that have oncogenic or tumor suppressive functions. For instance, if a miRNA is oncogenic, we would expect to see a two subsets of patients: one with no change in expression and one with high miRNA expression. We would not expect to see a subset with low expression. Conversely, if a miRNA is tumor suppressive, we would expect to see one set of patients with no change in expression and one with low miRNA expression, but we would not expect to see a subset with high expression.

3. **Line 85-89. The authors wrote "Bimodal miRNA were further confirmed by showing their usefulness in patient stratification based on overall survival and drug response (Figure 1B). After validating our methodology on breast cancer RNA-seq data". Validation results are not shown here. Figure 1B looks like just cartoon version of the workflow not the real results.**

Figure 1 describing the workflow has been moved to the introduction. Validation of the model using RNA-seq data has been added. We compare our method to 3 others and outline these results in the Results section, Table 1, and Figure 2.

4. **Line 92: "We applied the MK approach and found that cancer tissue had more bimodal miRNA than control (Figure 3A)." How much of the difference is due to smaller sample size in normal tissues? Can authors run the same model on each cancer data using a bootstrap of the same sample size as the normal tissue for each cancer?**

This analysis was redone using bootstrapped samples to account for differences in sample size between cancer and control datasets. This change is reflected in Figure 3 as well as the results.

5. **Line 96-97: "At a bimodality index ... " Once again, how much is that due to sample size differences and batch effects rather than cancer types?**

It does not appear that the number of bimodal miRNA is correlated with sample size:

| # Control Samples | # Bimodal miRNA |
| --- | --- |
| 105 | 56 |
| 33 | 37 |
| 50 | 34 |
| 59 | 30 |
| 91 | 28 |
| 44 | 25 |
| 45 | 24 |
| 104 | 14 |
| 52 | 11 |

| # Cancer Samples | # Bimodal miRNA |
| --- | --- |
| 835 | 56 |
| 861 | 37 |
| 372 | 34 |
| 506 | 30 |
| 997 | 28 |
| 523 | 25 |
| 446 | 24 |
| 1096 | 14 |
| 498 | 11 |

6. **Line 547: Supplementary Method file for model development and assessment is missing.**

The methods section has been revised to include a description of the datasets, mixture modeling, and validation. Additionally, validation of our method has been added as addressed in comment # 3.

Reviewer #1: 

7. **There are no descriptions of the parameters in Figure 1A.**

Figure 1 has been moved to the Introduction as a general workflow for the paper. A detailed description of the mixture model methodology has been added to the Methods section.

8. **In Figure 4A-H, there are no parameters described of the hierarchical clustering.**

Parameters for hierarchical clustering have been added as follows:

“Using all of the co-expression modules within each cancer type, hierarchical clustering was performed using Manhattan distance as the dissimilarity measure and complete linkage. Dendrograms were cut to yield two groups.”

9. **In Figure 4A-H, it would be great if you can switch group1 and group2 in some cancer types, for example, green for low expressed and purple for high expressed.**

This figure has been revised such that group 1 always has worse survival outcomes than group 2.

10. **Considering the heterogeneity of cancers, shall we focus on cancer specific bimodal miRNA?**

See response to comment #13.

Reviewer #2: 

**11. The abstract needs to be more coherent than the current version. The authors need to specify what motivates this work early in the abstract. Applying mixture models to study bimodal miRNA sounds like a logical step, but it is not clear to me what specific challenges they are trying to address and what they are hoping to find.**

The abstract has been revised to briefly include limitations of current statistical techniques and describe how the current study may identify novel oncogenic miRNA that can be utilized for personalized drug treatment (underlined below):

Heterogeneity of cancer means many tumorigenic genes are only aberrantly expressed in a subset of patients and thus follow a bimodal distribution, having two modes of expression within a single population. Traditional statistical techniques that compare sample means between cancer patients and healthy controls fail to detect bimodally expressed genes. We utilize a mixture modeling approach to identify bimodal microRNA (miRNA) across cancers, find consistent sources of heterogeneity, and identify potential oncogenic miRNA that may be used to guide personalized therapies. Functional pathway analysis was conducted to identify potential target genes of the bimodal miRNA. *In vivo* overexpression and knockout experiments were conducted to elucidate their clinical importance in chemotherapy treatments of cancer. In nine types of cancer, tumors consistently displayed greater bimodality than normal tissue. Specifically, in liver and lung cancers, high expression of miR-105 and miR-767 was indicative of poor prognosis. Functional pathway analysis identified target genes of miR-105 and miR-767 enriched in the phosphoinositide-3-kinase (PI3K) pathway, and analysis of over 200 cancer drugs *in vitro* showed that drugs targeting the same pathway show synergistic effects with high miR-105 and miR-767 levels. Overexpression of the two miRNA facilitated response to PI3K inhibitor treatment. We demonstrate that while cancer is marked by considerable genetic heterogeneity, there is between-cancer concordance regarding the particular miRNA that are more variable. Bimodal miRNA are ideal biomarkers that can be used to stratify patients for prognosis and drug response.

**12. A main conclusion of this manuscript is that “Bimodal miRNA are ideal biomarkers that can be used to stratify patients for prognosis and drug response”. However, Figures 3 I-P show that for many cancer types, bimodal miRNAs do not stratify patients. And is the purple group or the green group supposed to survive longer? These two groups swap between different cancer types. Can the authors explain that?**

The manuscript has been edited to in several places to more accurately reflect our findings, that bimodal miRNA are ideal biomarkers that can be used to stratify patients based on survival in several types of cancer. For figure 4, groups were assigned based on hierarchical clustering using different miRNA for each cancer type. Groups had been arbitrarily assigned names (either group 1 or group 2), but the figure has been revised such that group 1 has worse survival outcomes compared to group 2.

**13. Can the authors explain why they chose groups of bimodal miRNAs common to multiple cancer types to predict survival? They just mentioned that the estrogen receptor ESR1 and related miRNA may be bimodal in breast cancer, but they may not be bimodal in other cancer types.**

It is likely that there are cancer-specific bimodally expressed miRNA. Although we give the example of ESR1 in breast cancer, there are also several examples of genes that are mutated across cancer types (p53, PIK3CA, KRAS, MYC, etc.). Given that this is among the first studies to examine bimodal miRNA, we wanted to ensure that we focused on miRNA that had the greatest likelihood to contribute to tumorigenesis. Therefore, we chose bimodal miRNA that were common between cancer types. This explanation has been added to the manuscript:

We then focused on modules of bimodal miRNA that were common across cancers. Just as certain oncogenes and tumor suppressor genes widely contribute to tumorigenesis (e.g. p53, KRAS, PIK3CA, etc.), we hypothesized that particular miRNA broadly regulate cancer progression. Thus, we found four modules that were common to multiple cancer types.

**14. Figure 3A: what bimodality index was used for this analysis? I feel the analysis in Figure 3B should be done here to assess whether the results are consistent when you vary the BI threshold.**

Figure 3A was redone using bootstrapped samples. We report bimodal miRNA with BI > 0. This analysis was performed in order to broadly assess all sources of bimodality. We included the results when varying the BI threshold in the supplementary files.

Reviewer #3: 

Major:

15. **The authors mentioned the novel model development and assessment was detailed in the supplementary method file. However, unfortunately I was not able to access to it – seems missing, which is indeed essential to evaluate the method contribution of this manuscript. Also, as a computational biology paper, I would suggest to move the essential contents of the novel method to the main-text and instead move lots of experimental details to the supplementary. Couple of the following comments are related to the missing of the supplementary method file, but nevertheless I list them separately.**

The methods section has been revised to include a description of the datasets, mixture modeling, and validation. Additionally, the methods section has been formatted such that experimental details are now included in the supplementary methods.

16. **With the emphasis of their novel method and the first study to analysis bimodal miRNA, I would suggest the authors to perform simulations to demonstrate the advance of their new method over others regarding identify bimodal miRNAs with higher sensitivity and lower false positive rate, etc. Line 89, “After validating our methodology on breast cancer RNA-seq data,” – not clear to me whether the authors mentioned this methodology in the supplemental material? Or from their previous study (e.g. reference 7)? If the later, it will reduce the novelty of this manuscript regarding the new methodology although this is the first time applying on miRNA data.**

We have added validation of our method using breast cancer RNA-seq datasets. We compare our method to 3 others and outline these results in the Results section, Table 1, and Figure 2.

17. **It is not clear to me how many samples the authors used for assessing their models. I could guess the sample size from Figure 3, but please specify the details of analyzed samples in the genomic dataset section.**

Detailed description of the datasets has been added to the Methods section.

18. **Line 555, the authors used the top 3,000 predicted targeted genes for both miR-105 and miR-767. It sounds too many targeted genes which might include lots of false positives. I’m curious were the nine target genes (line 189-190) at the top list of the predicted putative targets? Were these nine genes validated using qPCR (e.g. included in the 12 significantly down regulated)? I would suggest the authors to double check how many of these 3,000 genes are actually expressed in corresponded cancers using RNA-Seq data downloaded from GDCs. Alternatively, maybe roughly 500 to 1000 genes would be a good option?**

In order to address the reviewer’s concern for potential false positives, the pathway analysis was performed using the top 1,000 gene targets instead of 3,000 per the reviewer’s suggestion. The analysis is included in Figure 6.

19. **Related to point above, I’m curious that, rather than performing a multi-step investigations on identifying direct targets that inhibit drug effects, why not the authors go validation with the common nine targets or 36 putative targets of both miR-105 and miR-767 (indicated in line 189) and investigate their potential roles regarding drug response. Any comments?**

We performed a pathway analysis using the top 1,000 target genes of miR-105 and miR-767 and found that the PI3K-Akt signaling pathway was significantly enriched. We then validated gene expression in the PI3K pathway and specifically looked at protein expression in the PI3K pathway in response to drug exposure.

20. **What’s the insight of the single bimodal miRNAs? Would be false positives? Or have different patterns regarding patient stratification?**

Single miRNA are likely true positives and could be analyzed for potential use in patient stratification. However, in the current study, we focused not only on patient stratification for survival, but we also wanted to capture miRNA that had functional roles in cancer and could be used to inform drug response. Therefore, we chose to look at groups of bimodal miRNA because these were more likely to have greater functional impact than single bimodal miRNA.

21. **If I understand/interpret the results of Figure S7 correctly, the expression levels are quite low for both miR-105 and miR-767. Would these levels of expression be sufficient to reflect the drug response? Again - please add the sample size information for the investigation between miRNA and drug response.**

Similar low miRNA expression levels have been observed by others using the same quantification method that we used (i.e. Taqman primers with U6 snRNA normalization) (<https://pubmed.ncbi.nlm.nih.gov/32493731/>, <https://pubmed.ncbi.nlm.nih.gov/23514736/>, <https://pubmed.ncbi.nlm.nih.gov/21784902/>). These studies describe functional consequences despite low expression. All cell experiments were performed in triplicate. Relationship between miRNA and drug response was examined in the13 cell lines.

22. **Given PI3K, AKT and mTOR are highly connected, how to determine PI3K is the direct therapeutic target?**

PI3K, AKT and mTOR are highly related and we looked at expression levels of several proteins involved all 3 pathways (e.g. AKT, IRS1, etc.) based on the of putative target of miR-105 and miR-767. Based on the RNA immunoprecipitation results, we found that *FOXO3*, *IRS1*, and *PDK1* were bound to AGO2 when the miRNA were overexpressed. Thus, we concluded that these three genes were likely direct targets of miR-105 and miR-767.

23. **Line 255, any particular reasons or interpretations on why the %cells in early apoptosis starts to decrease at 0.8 uM while %cells in late apoptosis continue increasing.**

A discussion of possible explanations has been added to the Results section:

It is unclear why the percentage of cells in early apoptosis decreased at the highest drug dosage (0.8 μM). It is possible that this dose immediately induced apoptosis, and while we were able to capture late apoptosis, we missed the maximal early apoptotic effect.

Minor:

24. **The resolution of the figures seems quite low even from the downloaded Tiff files as it look blurred when zooming in a bit. Please following the guideline of the journal and update the resolution.**

Figures have been uploaded with better resolution.

25. **Figure 8C, the y-axis go up to 60-100 fold changes which are extremely high. Would it be due to a very small expression level of the control condition?**

The high levels of p-AKT and p-ERK likely truly reflect the effects of insulin and ZSTK474, rather than low level in control. Insulin and ZSTK474 directly mediate the PI3K pathway. These effects have been observed previously in the literature (<https://pubmed.ncbi.nlm.nih.gov/12808134/>, <https://pubmed.ncbi.nlm.nih.gov/10669636/>, <https://pubmed.ncbi.nlm.nih.gov/21993922/>)

26. **Would the source codes become publically available (e.g. through GitHub)?**

Source codes will be made publicly available through GitHub (https://github.com/nutrigene/bimodality)

Reviewer #4:

Major:

27. **Supplementary method part is missing in the supplementary file, which makes the difficulty for the reviewer to judge their computational approach to calculate the bimodal index (in the first two paragraphs of the Method, they mentioned that the details will be described in supplementary method file…).**

Methods section has been revised to include detailed description of the bimodal index calculation. Results have been updated to reflect validation of our approach and comparison to other methodologies.

28. **How do they filter out the miRNA when the bimodal index is also high in general population? GTEx data is used? Again, this is not clear.**

This problem is not addressed by previous methods but was accounted for with our Controlled Mixture Modeling (CMK). An explanation of this has been added to the Methods section. In brief, if a miRNA was bimodally expressed in control samples, we incorporated a penalty into the calculation of the bimodality index. By penalizing the bimodality index calculation, we attempt to filter false positives when a miRNA is bimodally expressed in the control population.

29. **How do they determined the 1.4 cut off for bimodality index? It seems an arbitrary cut-off…**

Cutoff of 1.4 was determined based on prior studies that showed low level of false positives using that threshold. Previous studies tested bimodality indices between 1.1 and 1.5. They showed that a bimodality index threshold of 1.4 returns 0-1.2% false positives using a sample size of 50 and 0% false positives when the sample size is ≥ 200. This rationale has been added to the results section.

30. **How do they identify the co-expression module of bimodal miRNA? This is important for the following study.**

Description of the co-expression modules has been added:

Next, we examined the concurrent expression of bimodal miRNA. We postulated that modules of concurrently expressed miRNA would have a greater functional impact than single miRNA. Pearson correlation coefficients were calculated between miRNA pairs. Only miRNA with bimodality indices > 1.4 were considered. Groups of at least three miRNA were considered to be a module if all pairwise correlations were greater than 0.5.

31. **In Figure 4E, when cells were treated with miR-105 and miR-767 mimics, cell viability is increased. Will cell viability decrease when these two miRNA were inhibited?**

We did not perform knockdown experiments in the current study. While our results showed that miR-105 and miR-767 overexpression increased viability and apoptosis and altered expression in the PI3K pathway, we cannot conclude that these miRNA are *necessary* for cell viability or that PI3K genes are solely responsible for modulating drug effects. This is an important limitation of our study and excellent future direction that has been added to the Discussion section:

“Additional investigation should also aim to uncover the biological mechanisms by which miR-105 and miR-767 mediate drug sensitivity. We utilized computational tools to identify gene targets of miR-105 and miR-767 in the PI3K pathway. *In vitro* results also showed downregulation of IRS1 and FOXO3 with miR treatment, but without the appropriate knockdown experiments, we cannot conclude that these gene targets are directly responsible for increasing drug sensitivity. Since miR-105 and miR-767 have targets outside the PI3K pathway, it is possible that other genes may play a role. Future experiments should utilize Argonaute high-throughput sequencing of RNA isolated by crosslinking immunoprecipitation (AGO HITS-CLIP) to identify all miRNA-mRNA interactions [70]. Finally, other factors such as baseline gene expression and tumor microenvironment may mediate miRNA effects, so incorporating additional cell lines as well as in vivo studies will be crucial in finding the link between miR-105 and miR-767 and drug response.”

Minor:

**32. “in vitro” and “in vivo” should be italic.**

The manuscript has been edited to reflect this change.

**33. The resolution of figure within the main pdf is really low…**

See response to comment # 24.

**34. In the discussion, “Previous studies have simply removed known bimodal genes from the analysis”., please add citation here.**

Citation has been added.
