# Supplementary material for: Genome-wide cross-cancer analysis illustrates the critical role of bimodal miRNA in patient survival and drug responses to PI3K inhibitors
Source: PLoS Comput Biol. 2022 May 31;18(5):e1010109. doi: 10.1371/journal.pcbi.1010109 (PMC9187341; doi:10.1371/journal.pcbi.1010109)
Supplement: Attachment — Submitted filename: 20220127_Plos_Bimodal_Reviewers.docx [file pcbi.1010109.s018.docx]

**1. After checking the detailed methodology, it seems the authors tried to control the false positives by adjusting the BI only through penalizing those also showing bimodal in controls. This is an interesting idea. The authors reported the top bimodal genes (Figure 2) and the total number of genes at different BI thresholds (Table 1) to show the advance of their new CMK method. However, I assume it would be more interesting to investigate examples that were detected by CMK but not MK to illustrate the contribution of introducing control samples. In addition, later on when applying the CMK on identifying bimodal miRNAs, the authors further emphasized to focus on those were common across cancers. I’m curious that whether or not considering the common bimodal miRNAs detected in cancers only would already compensate the risk of bimodal miRNAs in controls?**

We have added a discussion of the genes identified by mixture modeling but excluded when accounting for control samples. Specifically, we determined that at BI > 1, the genes excluded by CM contained genes that were known to have high copy number variation and sequence variation in the general population. We also show that excluded genes indeed have similar distributions in cancer and control samples and added these examples to figure 2D. We have included a description of this in the results section:

“Additionally, we visualized genes that were identified by MM but excluded by CM at a BI > 1. These genes had distributions that were very similar between cancer and control (**Figure 2D**). Of the 100 genes with BI > 1 that were identified by MM but excluded by CM, 33 coded for immunoglobulin chains. This finding is reflective of the high sequence variation observed in these genes within the general population. Another gene excluded by CM at BI > 1 was glutathione S-transferase mu 1 (*GSTM1*) which is known to have variable enzymatic activity in the general population due to polymorphic deletions.”

It is possible that identifying bimodal miRNA across cancers would account for risk of bimodal control miRNA being identified. However, there may be genes that are not accounted for using this strategy. For example, sex-bias miRNA or miRNA that are expressed across tissue types might still be identified despite looking for similarities across cancers.

**2. Line 119-120, the authors examined the known bimodal genes in breast cancer: ERS1, HER2 and PGR. Please add relevant references for these three bimodal genes.**

This citation has been added (see line 113).

**3. Line 123, “which ranked it 21st (Table 1)”. Please correct this typo.**

This typo has been corrected.

**4. The resolution of figures seems still low (i.e. 150 dpi).**

This mistake has been corrected and figures have been submitted with 600 dpi.

**5. The authors provided a GitHub repository link for the source codes but currently unavailable to assess.**

Codes used to calculate bimodality index, generate histograms, and plot survival curves have been deposited in GitHub (<https://github.com/nutrigenelab/bimodality>) .
